# Case report: Meningioma associated with meningioangiomatosis mimicking invasive meningioma

**DOI:** 10.3389/fneur.2023.1200827

**Published:** 2023-06-28

**Authors:** Rong Ge, Jun Yang, Xiangang Yin, Jingya Wang

**Affiliations:** ^1^Ningbo Clinical Pathology Diagnosis Center, Ningbo, China; ^2^Surgery Center, The Affiliated People’s Hospital of Ningbo University, Ningbo, China

**Keywords:** meningioangiomatosis, meningioma, invasive meningioma, pathology, neurology

## Abstract

Meningioangiomatosis (MA) is a rare malformation or hamartomatous lesion in the central nervous system, characterized by a plaque-like mass within the leptomeninges and cerebral cortex. An even rarer condition is MA complicated with meningiomas. We herein report a case of meningioma associated with MA that might be erroneously interpreted as a higher-grade lesion or an invasion by preoperative radiologic and postoperative histological examinations.

## Introduction

Meningioangiomatosis (MA) features vascular proliferation and perivascular cell growth (1). However, the pathogenesis of MA remains unclarified, although it was originally described as a condition associated with neurofibromatosis (NF)-2 (2). The principal hypotheses include hamartomatous lesions, direct invasion of malignant meningiomas, and cortical vascular malformations (1). Meningiomas combined with MA (MA-M) are extremely rare (3). MA-M may often show similar radiologic and histological results to an invasive meningioma, which presents challenges to the treatment (4). To further the understanding of this disorder, we present a case of MA-M in a 28 years-old man and discuss its clinical presentation and histopathologic characteristics.

## Case presentation

**Figure 1:**
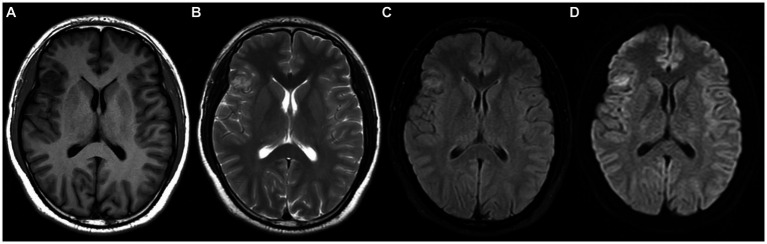
Magnetic resonance imaging (MRI) shows a heterogeneously enhanced intradural lesion in the right frontotemporal lobe with dural enhancement, extending to the superficial cortex. **(A)** T1W with contrast, **(B)** T2W, **(C)** T2W flair, **(D)** DWI.

A 28 years-old man presented with maxillofacial discomfort and headache for 2 weeks, and then visited a regional hospital for consultation. Neurologic and physical exams showed he was both mentally and physically healthy. No past medical history was reported. Computer tomography (CT) indicated a high-density nodule in the right frontal lobe. Magnetic resonance imaging (MRI) with contrast revealed a heterogeneously intradural lesion measuring 19 × 17 cm in the right frontotemporal lobe (Figure 1). A meningioma or an oligodendroglioma was suspected.

The patient underwent the conventional right pterional craniotomy, which revealed an ill-defined mass with a soft grayish-white surface. There were many calcifications in the middle, and the surrounding tissue was slightly edematous. The tumor tissue was completely resected under the microscope.

**Figure 2:**
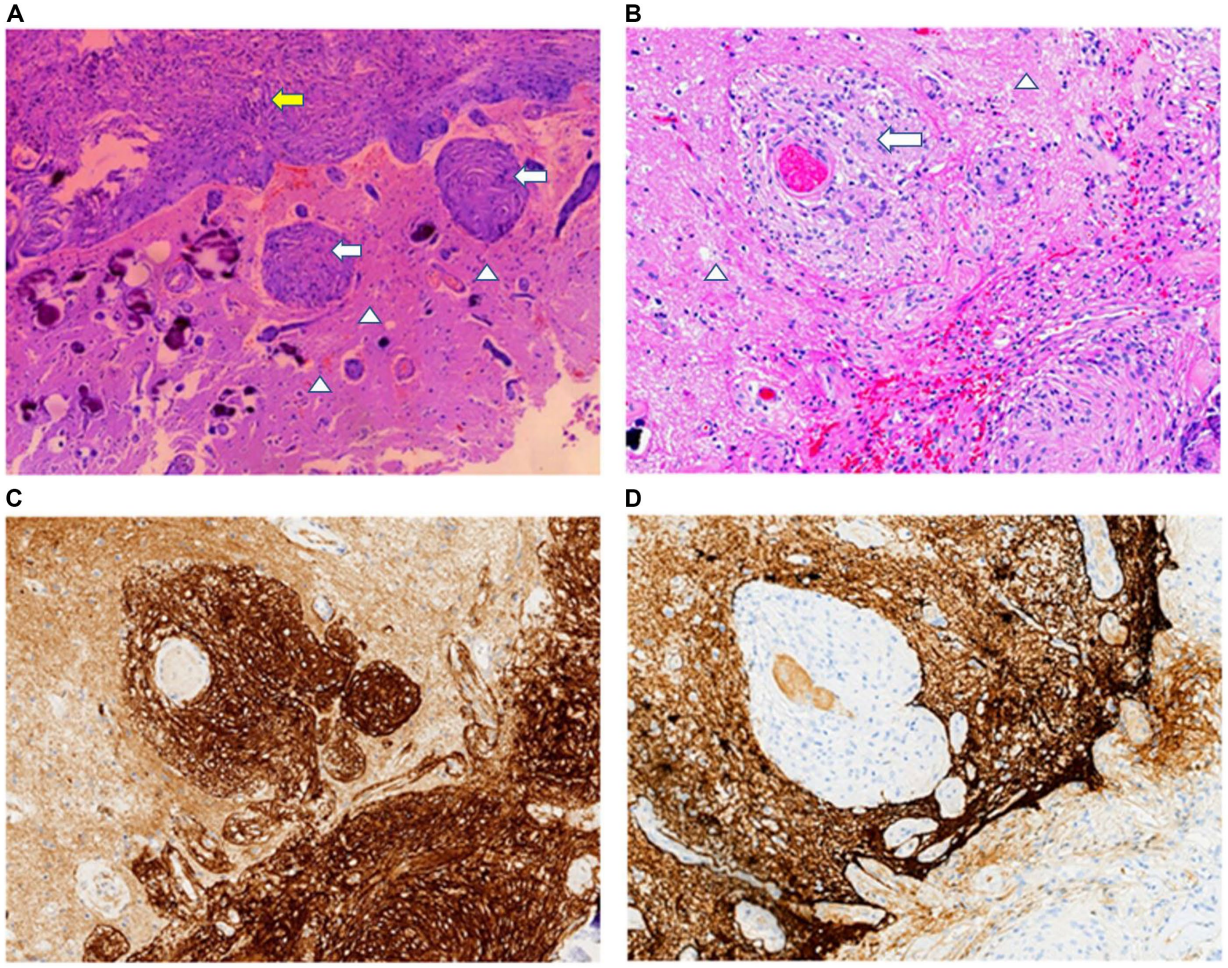
**(A)** In the transition area between the normal brain (arrowheads) and meningioma (yellow arrow), abnormal small blood vessels and scattered psammomas can be observed. **(B)** MA area is typical of small blood vessels cuffed by meningothelial cells (white arrows) or fibroblasts proliferation. Tumor cells and perivascular meningothelial or fibroblast-like spindle cells are positive for SSTR2 **(C)** but lack immunoreactivity for GFAP **(D)**. H&E staining: **A** × 40; **B** × 100. Immunohistochemical staining: **C**,**D** × 100.

Microscopically, the lesion was composed of meningothelial and spindle cells, which formed small whorls. In the whorl center, psammoma bodies were observed. There was no evidence of necrosis or mitotic activity. The histopathological features of the lesion were consistent with those of a transitional meningioma. In the adjacent cerebral parenchyma to the lesion, significant proliferation of meningothelial cells surrounding the small blood vessels was detected (Figures 2A,B). This was the same as the characteristic morphology of MA. Immunohistochemical analysis revealed that tumor cells of the meningioma were positive for vimentin, somatostatin receptor (SSTR)-2 (Figure 2C), epithelial membrane antigen (EMA), and progesterone receptor (PR). The perivascular fibroblast-like spindle cells were positive for vimentin and SSTR2 but lacked immunoreactivity for EMA and GFAP (Figure 2D). Ki-67 (MIB-1) index was smaller than 2% in all areas. Mutational analysis of NF2 was negative.

Based on the above results, the patient was finally diagnosed with a transitional meningioma (WHO grade 1) associated with MA. After surgery, the patient was followed up for 8 months without receiving any adjuvant radiotherapy or chemotherapy. During this period, the patient was disease-free.

### Literature review

All English literature about MA-M published on PubMed was reviewed, and the clinical pathology information on sex, age, location of the lesion, clinical presentation, histopathological type of the meningioma, and clinical outcomes was obtained. A total of 62 cases of MA-M were included. Clinical information on these cases and the present case is detailed in Table 1 (5–25).

**Table 1:** Case reports and series of MA-M in the English literature.

Author (year)	*N*/sex	Age	Presentation	Location	Histopathologic type of meningioma	Outcome
Auer et al. (5)	1/M	15	Subarachnoid hemorrhage	Frontal lobe	Fibrous	Died from complications
Louw et al. (6)	2/M	15–33	Subarachnoid hemorrhage, headache	Frontal lobe	Fibrous, transitional	NA
Blumenthal et al. (7)	1/M	0.9	Seizure	Frontal lobe	Transitional	NR
Giangaspero et al. (8)	2/M	9–28	Asymptomatic, seizure	Temporal, frontal lobe	Transitional	NR
Mut et al. (9)	1/F	20	Seizure	Temporal lobe	Transitional	NR
Sinkre et al. (10)	1/M	8	Headache	Frontal lobe	Atypical	NR
Kim et al. (11, 12)	6/M, 2/F	3–19	Seizure, headache	Frontoparietal, temporal, frontal, parieto-occipital lobe	Fibrous, transitional, meningothelial, psammomatous	NR, recurrence (1 case)
Iezza et al. (13)	1/M	33	Seizure	Frontal lobe	Transitional	NA
Kuchelmeister et al. (14)	1/M	58	Headache and forgetfulness	Frontal lobe	Microcystic	NR
Perry et al. (3)	8/M, 2/F	0.8–35	NA	Frontal, temporal lobe	Transitional, meningothelial, atypical	NA
Deb et al. (15)	1/F	1.5	Seizure	Temporal lobe	Transitional	NR
Saad et al. (16)	1/F	3	Seizure	Frontal lobe	NA	NA
Shi et al. (17)	1/F	50	Headache and dizziness	Temporal lobe	Meningothelial	NR
Chen et al. (18)	1/M	34	Numbness and weakness of lower extremity	Frontoparietal lobe	Atypical	NR
Cui et al. (19)	1/M	33	Seizure	Frontal lobe	Transitional	NR
Zhang et al. (20)	7/M	3–32	Headache, seizure, diabetes insipidus	Frontal, temporal, parietal lobe, corpus callosum, third ventricle	Transitional, fibrous, psammomatous	NR, died (1 case)
Diao et al. (21)	1/F	16	Seizure	Parieto-occipital lobe	Meningothelial	NR
Galloway et al. (22)	1/M	1.5	Seizure	Temporal lobe	Rhabdoid	NR
Hassan et al. (4)	1/M	11	Headache and diplopia	Temporal lobe	Atypical	NR
Dono et al. (23)	1/F, 1/M	14–26	Seizure	Temporal lobe	NA	NR
Liu et al. (24)	1/M	8	Dizziness and seizure	Parietal lobe	Fibrous	NR
Zhang et al. (25)	9/M, 7/F	3–46	Seizure, headache, nausea, organic psycho-syndrome, hyperhidrosis and hearing loss, facial paralysis, polyuria	Frontal, parietal, temporal lobe, sphenoid ridge, anterior cranial fossa, callosum, third ventricle	Fibrous, transitional, meningothelial, atypical, metaplastic	NR, died (1 case)
Present	1/M	28	Maxillofacial discomfort and headache	Frontotemporal lobe	Transitional	NR

NR, no recurrence; NA, not available.

## Discussion

MA has been described to occur together with meningiomas, oligodendrogliomas, schwannomas, encephaloceles, and vascular malformations in rare cases (3). Among these co-morbidities, MA associated with meningiomas is the commonest (19). At the time of writing, we had found 63 cases of MA-M including the present case in all the English literature reviewed (5–25). There were 46 men and 17 women (male to female ratio, 2.7:1). MA-M tended to occur in young patients with a mean age of 17.5 years (0.8 to 58 years). The most common symptom were seizures (31 cases) and headache (11 cases). The lesion mainly affected the frontal lobe (23 cases) and temporal lobe (18 cases).

Radiologically, pre-surgical diagnosis of MA-M is challenging due to its variable appearances. MRI is usually the imaging modality of choice, and MA-M lesions typically appear hypo-or iso-intense on T1-weighted images and hyperintense on T2-weighted images (24). MRI results showed that the majority of the MA-M cases had intracortical non-enhanced lesions without dural attachment.

Transitional (23 cases) and fibroblastic (14 cases) meningiomas were the predominant subtypes in the MA-M patients. Additionally, there were also 8 atypical, 7 meningothelial, 4 metaplastic, 2 psammomatous, 1 microcystic, and 1 rhabdoid subtypes. A clear transition zone between neoplastic meningiomas and MA was not found. Similar to MA, the adjacent cerebral parenchyma was also histopathologically characterized by cortical meningovascular proliferation and the perivascular spread of meningothelial and fibroblast-like cells along the Virchow–Robin spaces (19). Neither necrosis nor mitotic activity was detected. Immunohistochemistry was of small diagnostic value, as staining patterns were inconsistent among cases (25). The Ki-67 labelling index values ranged from <1% to 15%, most of which were less than 1% (12). The diagnosis and classification of meningioma rely on analyzing both its histopathological features and genetic alterations (26). The WHO grade of MA-A is determined by the grade of the accompanying meningioma.

The pathogenesis of MA requires further investigation. A few hypotheses on MA pathogenesis have been proposed, including developmental and hamartomatous lesions, cortical vascular malformations, and direct invasion into the brain parenchyma by a meningioma (1). Among these hypotheses, the hamartomatous or cortical dysplastic etiology were favored based on the lack of significant proliferative activity and benign clinical course in most MA cases.

MA-M can be easily mistaken for invasive meningiomas because of its feature of cortical invasion (4). Meningothelial and vascular proliferation along the Virchow–Robin spaces do not actually represent parenchymal invasion and have no direct relationship with the risk of recurrence (1). True invasion takes place when tumor cells break through the pia mater to invade the cerebral parenchyma. Generally, there is a serrated irregular outer border intermixed with the cerebral cortex in invasive meningiomas. Unlike invasive meningiomas, MA-M is benign, lacking such features as atypia, mitoses, and necrosis, with an excessively low value of Ki-67 labelling index.

Surgery resection is the primary treatment for MA-M and the surgical approach is similar to that for invasive meningioma. Incomplete resection of MA-M might pose a risk of recurrence or epilepsy. Surgical resection is the recommended treatment for recurrent tumors that have undergone prior excision, with adjuvant radiotherapy being considered in selected cases (27). None of the studied patients received radiotherapy and chemotherapy after gross-total resection. Follow-up information of 49 cases was available, and the follow-up time ranged from 5 to 113 months. At the end of the follow-up, 45 patients were alive with no evidence of disease, 1 patient suffered recurrence (11), and 3 patients died of operative complications or other diseases (12, 20, 25).

Due to the good prognosis of MA-M, more precise diagnosis for these rare cases is important, which provides guidance for clinical management. Preclinical models of meningioma may provide an ideal platform for testing the molecular mechanisms of tumor development and targeted therapies (28). Advancements in artificial intelligence and radiomics have facilitated the characterization of meningiomas, providing critical information for this disease.

## Data availability statement

The original contributions presented in the study are included in the article/supplementary material, further inquiries can be directed to the corresponding author.

## Ethics statement

The studies involving human participants were reviewed and approved by Ningbo Clinical Pathology Diagnosis Center. The patients/participants provided their written informed consent to participate in this study. Written informed consent was obtained from the individual(s) for the publication of any potentially identifiable images or data included in this article.

## Author contributions

RG: collection of the data and drafting the manuscript. JY and XY: data analysis and statistical analysis. JW: critical revisions of the manuscript. All authors contributed to the article and approved the submitted version.

## Funding

This study was supported by the Medical and Health Research Project of Zhejiang Province (2022KY1185) and Ningbo Leading Medical & Health Discipline (2022-F30).

## Conflict of interest

The authors declare that the research was conducted in the absence of any commercial or financial relationships that could be construed as a potential conflict of interest.

## Publisher’s note

All claims expressed in this article are solely those of the authors and do not necessarily represent those of their affiliated organizations, or those of the publisher, the editors and the reviewers. Any product that may be evaluated in this article, or claim that may be made by its manufacturer, is not guaranteed or endorsed by the publisher.
